# (6*S*)-2-*tert*-Butyl-6-[(4*S*,5*R*)-3,4-dimethyl-5-phenyloxazolidin-2-yl]phenol

**DOI:** 10.1107/S1600536810009190

**Published:** 2010-03-24

**Authors:** Alexander E. Anderson, Kate L. Edler, Raleigh W. Parrott, Shawn R. Hitchcock, Gregory M. Ferrence

**Affiliations:** a CB 4160, Department of Chemistry, Illinois State University, Normal, IL 61790, USA

## Abstract

The title compound, C_21_H_27_NO_2_, exhibits hydrogen bonding between the phenolic H atom and the heterocyclic N atom. The absolute configuration of the mol­ecule is known from the synthetic procedure.

## Related literature

For related structures and background to the use of chiral oxazolidines as templates in asymmetric synthesis, see: Agami & Couty (2004 ▶); Campbell *et al.* (2010 ▶); Koyanagi *et al.* (2010 ▶); Parrott & Hitchcock (2007 ▶); Parrott *et al.* (2008 ▶). The synthesis of the title compound is described by Parrott & Hitchcock (2007 ▶). The absolute configuration assignment is based on both optical activity measurements and on the known stereochemistry of the commercially obtained optically pure ephedrine from which it was prepared (Parrott & Hitchcock, 2007 ▶). For geometry checks using *Mogul*, see: Bruno *et al.* (2004 ▶). For ring puckering analysis, see: Boeyens (1978 ▶); Cremer & Pople (1975 ▶); Spek (2009 ▶).  For a description of the *Jmol* toolkit for the preparation of enhanced figures, see: McMahon & Hanson (2008 ▶).
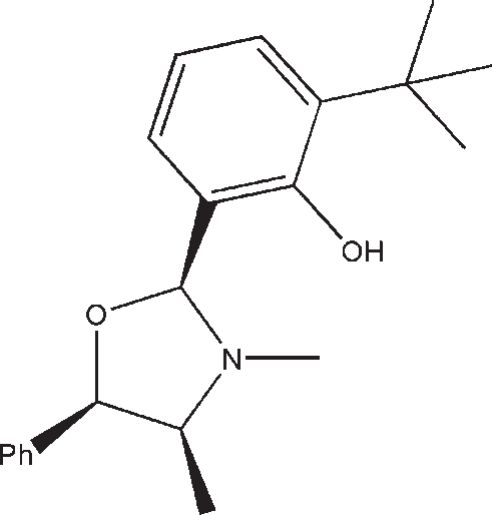


## Experimental

### 

#### Crystal data

C_21_H_27_NO_2_
*M* *_r_* = 325.44
Monoclinic, 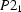

*a* = 8.3288 (8) Å
*b* = 9.8657 (9) Å
*c* = 11.4325 (11) Å
β = 91.667 (1)°
*V* = 939.00 (15) Å^3^
*Z* = 2
Mo *K*α radiation
μ = 0.07 mm^−1^
*T* = 140 K
0.55 × 0.27 × 0.27 mm

#### Data collection

Bruker SMART APEX CCD diffractometer
Absorption correction: multi-scan (*APEX2*; Bruker, 2008 ▶) *T*_min_ = 0.687, *T*_max_ = 0.746
9040 measured reflections
2284 independent reflections
2191 reflections with *I* > 2σ(*I*)
*R*_int_ = 0.017

#### Refinement

*R*[*F* ^2^ > 2σ(*F* ^2^)] = 0.028
*wR*(*F* ^2^) = 0.074
*S* = 1.04
2284 reflections
221 parameters
1 restraint
H atoms treated by a mixture of independent and constrained refinement
Δρ_max_ = 0.21 e Å^−3^
Δρ_min_ = −0.14 e Å^−3^

### 

Data collection: *APEX2* (Bruker, 2008 ▶); cell refinement: *SAINT* (Bruker, 2008 ▶); data reduction: *SAINT*; program(s) used to solve structure: *SIR2004* (Burla *et al.*, 2005 ▶); program(s) used to refine structure: *SHELXL97* (Sheldrick, 2008 ▶); molecular graphics: *ORTEP-3 for Windows* (Farrugia, 1997 ▶); software used to prepare material for publication: *WinGX* (Farrugia, 1999 ▶), *publCIF* (McMahon & Westrip, 2008 ▶) and *Mercury* (Macrae *et al.*, 2008 ▶).

## Supplementary Material

Crystal structure: contains datablocks global, I. DOI: 10.1107/S1600536810009190/zl2269sup1.cif

Structure factors: contains datablocks I. DOI: 10.1107/S1600536810009190/zl2269Isup2.hkl

Additional supplementary materials:  crystallographic information; 3D view; checkCIF report

**Fig. 2.**
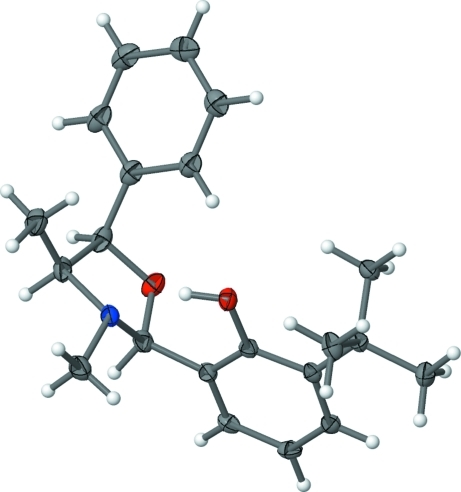
The enhanced Jmol figure of the title compound. This is the third in a series of three Jmol figures intended to illustrate some versatility of the program. See also: Campbell et al. (2010); Koyanagi et al. (2010). In this Jmol, all interactive features are defined by text editing or hand writing the scripts. Most script artifacts are resolved by using this method and the orientation of the molecule only depends upon the end-user's preference.

Enhanced figure: interactive version of Fig. 2

## Figures and Tables

**Table 1:** Hydrogen-bond geometry (Å, °)

*D*—H⋯*A*	*D*—H	H⋯*A*	*D*⋯*A*	*D*—H⋯*A*
O20—H20⋯N3	0.90 (2)	1.79 (2)	2.6244 (16)	154.4 (19)

**Fig. 1.**
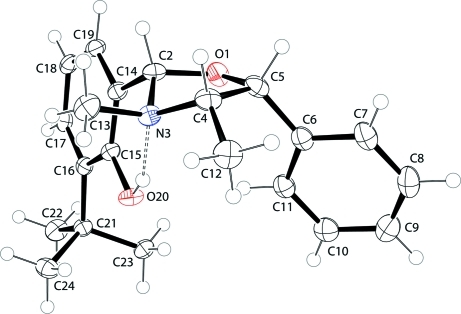
The molecular structure of the title compound with the atomic numbering scheme. The intramolecular H-bonding is denoted by the dashed line and the 3E heterocycle conformation is shown. Displacement ellipsoids are drawn at the 50% probability level and H-atoms are shown as spheres of arbitrary size.

## supplementary crystallographic information

### Comment

Chiral oxazolidines are useful templates for conducting asymmetric syntheses (Agami & Couty, 2004). In order to explore the utility of these compounds in the catalytic asymmetric addition of diethylzinc to aldehydes, we prepared a series of oxazolidines from (1*R*,2*S*)-ephedrine, (1*S*,2*S*)-pseudoephedrine (Parrott & Hitchcock, 2007) and (1*R*,2*S*)-norephedrine (Parrott *et al.*, 2008). In the course of synthesizing these oxazolidines, we were able to obtain crystals suitable for X-ray crystallographic analysis.

The title compound is structurally similar to two other oxazolidines published by Koyanagi *et al.* (2010) and Campbell *et al.* (2010). Despite these structural similarities, a *Mogul* geometry check (Bruno *et al.*, 2004) reveals differences between the three structures. The C2—N3—C4 angle is found to be usual in the title compound (103.0 (1)°) and in Koyanagi *et al.* (106.0 (1)°), but not in Campbell *et al.* (106.2 (1)°), even though the last two angles are not significantly different. Another angle, C5—O1—C2, considered unusual in *Mogul* for both Koyanagi *et al.* and Campbell *et al.* (103.9 (1)° and 103.3 (1)°, respectively) is not unusual in the title compound (108.9 (1)°). These differences could be due to the difference in substituents on the N3 position in these compounds. The methyl substituent in the title compound occupies less space than the isopropyl substituent in the other two structures, allowing for more typical bond angles in the heterocycle of the title compound.

The difference in bond angles also appears in *PLATON* in the ring-puckering analysis of these compounds (Spek, 2009; Cremer & Pople, 1975; Boeyens, 1978). Koyanagi *et al.* and Campbell *et al.* report O1—C2—N3—C4—C5 ring conformations close to ^1^E (Φ = 1.63 (17)° and -7.05 (19)°, respectively), with O1 as the flap apex, while the heterocycle conformation of the title compound is closer to an ^3^E conformation (Φ = 65.73 (20)°), resulting in N3 as the flap apex. Even with these differences, the distance between the hydrogen donor and acceptor in all three structures is virtually identical (2.6244 Å in the title compound compared to 2.6278 (16) Å and 2.6180 (17) Å in Koyanagi *et al.* and Campbell *et al.*, respectively).

About the *Jmol* enhanced figure:

We are reporting three related structures containing *Jmol* enhanced figures, one in this paper and the other two in other papers in this *Journal* (Campbell *et al.*, 2010; Koyanagi *et al.*, 2010). The *Jmol* enhanced figures were created to illustrate a range of author convenience versus end user experience, ranging from a purely GUI driven experience for the author resulting in a less functional figure for the end user to a more sophisticated use of the *Jmol* scripting by the author resulting in a more polished and versatile figure for the end user. The buttons, check boxes and radio buttons in the three examples visually appear to be identical; however, the underlying code they execute results in significantly different overall responses by the *Jmol* visualizer.

The *Jmol* enhanced figure included with this paper required substantial author hand-coding of *Jmol* scripts. To generate this enhanced figure, substantial author familiarity with *Jmol* script coding is required and generation of the figure is significantly more time-consuming. Strictly authoring with the *Jmol* toolkit GUI, without text editing any code, provides a relatively quick and easy means to prepare a decent enhanced figure, and is often sufficient. For advanced users, hand-coding *Jmol* scripts provides a much more versatile figure for the end-user. In particular, the orientation information of the structure can be eliminated from all radio buttons, buttons, and check boxes. This becomes a major advantage when the end-user toggles the figure to rotate or changes the orientation from the location dictated in the script. When a new option is selected, the figure will only change in the areas being highlighted. Another advantage of stripping the script of lines not related to the defined option is the ability to see more than one option at a time. For instance, in this enhanced figure, the thermal ellipse color may be viewed even when the atom style is not in the ellipse mode.

### Experimental

The title compound was synthesized as previously reported (Parrott & Hitchcock, 2007). Single crystals were grown by vapor diffusion of hexane into a methylene chloride solution of the title compound.

### Refinement

All non-H atoms were refined anisotropically without disorder. The absolute configuration assignment is based on both optical activity measurements and on the known stereochemistry of the commercially obtained optically pure ephedrine from which it was prepared (Parrott & Hitchcock, 2007). All H atoms were initially identified through difference Fourier syntheses and then, except for the O–H hydrogen atom, removed and included in the refinement in the riding-model approximation (C–H = 0.95, 0.98, and 1.00 Å for Ar–H, CH_3_ and CH; U_iso_(H) = 1.2U_eq_(C) except for methyl groups, where U_iso_(H) = 1.5U_eq_(C)). The OH H atom was freely refined isotropically. In the absence of significant anomalous scattering effects, Friedel pairs were merged.

### Crystal data

**Table:**

C_21_H_27_NO_2_	*F*(000) = 352
*M**_r_* = 325.44	*D*_x_ = 1.151 Mg m^−^^3^
Monoclinic, *P*2_1_	Mo *K*α radiation, λ = 0.71073 Å
Hall symbol: P 2yb	Cell parameters from 5731 reflections
*a* = 8.3288 (8) Å	θ = 2.5–3.4°
*b* = 9.8657 (9) Å	µ = 0.07 mm^−^^1^
*c* = 11.4325 (11) Å	*T* = 140 K
β = 91.667 (1)°	Rod, colourless
*V* = 939.00 (15) Å^3^	0.55 × 0.27 × 0.27 mm
*Z* = 2	

### Data collection

**Table:**

Bruker SMART APEX CCD diffractometer	2191 reflections with *I* > 2σ(*I*)
graphite	*R*_int_ = 0.017
ω scans	θ_max_ = 27.5°, θ_min_ = 1.8°
Absorption correction: multi-scan (*APEX2*; Bruker, 2008)	*h* = −10→10
*T*_min_ = 0.687, *T*_max_ = 0.746	*k* = −12→12
9040 measured reflections	*l* = −14→14
2284 independent reflections	

### Refinement

**Table:**

Refinement on *F*^2^	Primary atom site location: structure-invariant direct methods
Least-squares matrix: full	Secondary atom site location: difference Fourier map
*R*[*F*^2^ > 2σ(*F*^2^)] = 0.028	Hydrogen site location: inferred from neighbouring sites
*wR*(*F*^2^) = 0.074	H atoms treated by a mixture of independent and constrained refinement
*S* = 1.04	*w* = 1/[σ^2^(*F*_o_^2^) + (0.0485*P*)^2^ + 0.090*P*] where *P* = (*F*_o_^2^ + 2*F*_c_^2^)/3
2284 reflections	(Δ/σ)_max_ < 0.001
221 parameters	Δρ_max_ = 0.21 e Å^−^^3^
1 restraint	Δρ_min_ = −0.14 e Å^−^^3^

### Special details

**Table:**

Geometry. All s.u.'s (except the s.u. in the dihedral angle between two l.s. planes) are estimated using the full covariance matrix. The cell s.u.'s are taken into account individually in the estimation of s.u.'s in distances, angles and torsion angles; correlations between s.u.'s in cell parameters are only used when they are defined by crystal symmetry. An approximate (isotropic) treatment of cell s.u.'s is used for estimating s.u.'s involving l.s. planes.
Refinement. Refinement of *F*^2^ against ALL reflections. The weighted *R*-factor wR and goodness of fit *S* are based on *F*^2^, conventional *R*-factors *R* are based on *F*, with *F* set to zero for negative *F*^2^. The threshold expression of *F*^2^ > 2σ(*F*^2^) is used only for calculating *R*-factors(gt) etc. and is not relevant to the choice of reflections for refinement. *R*-factors based on *F*^2^ are statistically about twice as large as those based on *F*, and *R*- factors based on ALL data will be even larger.

### Fractional atomic coordinates and isotropic or equivalent isotropic displacement parameters (Å^2^)

**Table:**

	*x*	*y*	*z*	*U*_iso_*/*U*_eq_	
O1	0.99692 (12)	0.27815 (11)	0.41600 (9)	0.0248 (2)	
O20	0.65433 (12)	0.17836 (11)	0.28853 (9)	0.0213 (2)	
N3	0.79655 (14)	0.40974 (12)	0.33736 (10)	0.0202 (2)	
C16	0.75996 (15)	0.03243 (13)	0.14241 (11)	0.0174 (3)	
C14	0.91785 (16)	0.22492 (14)	0.21866 (11)	0.0185 (3)	
C19	1.04036 (16)	0.19395 (15)	0.14295 (12)	0.0204 (3)	
H19	1.1354	0.2472	0.1438	0.025*	
C2	0.94417 (16)	0.33629 (15)	0.30751 (12)	0.0210 (3)	
H2	1.0265	0.4016	0.2795	0.025*	
C6	0.87041 (16)	0.27789 (16)	0.60254 (12)	0.0233 (3)	
C15	0.77689 (15)	0.14637 (14)	0.21694 (11)	0.0177 (3)	
C4	0.84147 (17)	0.47509 (15)	0.45020 (13)	0.0241 (3)	
H4	0.9104	0.5556	0.4342	0.029*	
C21	0.61341 (16)	−0.06087 (14)	0.14940 (12)	0.0189 (3)	
C23	0.60832 (17)	−0.12120 (14)	0.27378 (12)	0.0221 (3)	
H23A	0.515	−0.1811	0.2791	0.033*	
H23B	0.6	−0.0477	0.3309	0.033*	
H23C	0.7067	−0.1731	0.2903	0.033*	
C8	0.71138 (18)	0.08617 (17)	0.66066 (14)	0.0280 (3)	
H8	0.6469	0.0099	0.6402	0.034*	
C22	0.62164 (19)	−0.17981 (16)	0.06338 (13)	0.0273 (3)	
H22A	0.526	−0.2368	0.0706	0.041*	
H22B	0.718	−0.2338	0.0813	0.041*	
H22C	0.6261	−0.1448	−0.0167	0.041*	
C17	0.88590 (16)	0.00653 (14)	0.06749 (11)	0.0199 (3)	
H17	0.8768	−0.0681	0.0152	0.024*	
C13	0.7473 (2)	0.50590 (16)	0.24528 (14)	0.0285 (3)	
H13A	0.6492	0.5527	0.2679	0.043*	
H13B	0.7267	0.4568	0.1718	0.043*	
H13C	0.833	0.5724	0.2345	0.043*	
C9	0.74020 (19)	0.11793 (18)	0.77790 (14)	0.0316 (4)	
H9	0.6968	0.0629	0.8374	0.038*	
C5	0.94712 (16)	0.36461 (16)	0.51019 (12)	0.0242 (3)	
H5	1.0446	0.4091	0.546	0.029*	
C18	1.02423 (16)	0.08575 (15)	0.06636 (12)	0.0215 (3)	
H18	1.1069	0.0657	0.0135	0.026*	
C11	0.89769 (18)	0.30989 (18)	0.72035 (13)	0.0284 (3)	
H11	0.9612	0.3866	0.7412	0.034*	
C24	0.45695 (17)	0.01669 (16)	0.11992 (14)	0.0257 (3)	
H24A	0.3653	−0.0452	0.1249	0.039*	
H24B	0.4612	0.0534	0.0404	0.039*	
H24C	0.4447	0.0912	0.1757	0.039*	
C10	0.8326 (2)	0.23041 (19)	0.80740 (14)	0.0323 (4)	
H10	0.8516	0.2532	0.8874	0.039*	
C12	0.69710 (19)	0.52203 (17)	0.51714 (14)	0.0301 (3)	
H12A	0.6389	0.5915	0.4717	0.045*	
H12B	0.7333	0.5601	0.5926	0.045*	
H12C	0.6259	0.4448	0.5304	0.045*	
C7	0.77616 (17)	0.16510 (16)	0.57324 (13)	0.0244 (3)	
H7	0.7564	0.1424	0.4934	0.029*	
H20	0.680 (2)	0.258 (3)	0.3212 (18)	0.040 (5)*	

### Atomic displacement parameters (Å^2^)

**Table:**

	*U*^11^	*U*^22^	*U*^33^	*U*^12^	*U*^13^	*U*^23^
O1	0.0234 (5)	0.0272 (5)	0.0237 (5)	0.0053 (4)	−0.0006 (4)	−0.0053 (4)
O20	0.0187 (4)	0.0178 (5)	0.0278 (5)	−0.0037 (4)	0.0079 (4)	−0.0053 (4)
N3	0.0219 (5)	0.0147 (5)	0.0239 (6)	−0.0007 (4)	0.0028 (4)	−0.0002 (4)
C16	0.0189 (6)	0.0150 (6)	0.0183 (6)	0.0009 (5)	−0.0004 (5)	0.0027 (5)
C14	0.0196 (6)	0.0160 (6)	0.0201 (6)	−0.0004 (5)	0.0022 (5)	0.0015 (5)
C19	0.0175 (6)	0.0212 (7)	0.0227 (6)	−0.0013 (5)	0.0028 (5)	0.0050 (5)
C2	0.0182 (6)	0.0191 (6)	0.0259 (7)	−0.0040 (5)	0.0032 (5)	−0.0010 (5)
C6	0.0200 (6)	0.0257 (7)	0.0242 (7)	0.0065 (6)	−0.0015 (5)	−0.0031 (6)
C15	0.0181 (6)	0.0156 (6)	0.0195 (6)	0.0014 (5)	0.0031 (5)	0.0016 (5)
C4	0.0251 (7)	0.0172 (6)	0.0303 (7)	−0.0042 (5)	0.0023 (5)	−0.0051 (5)
C21	0.0204 (6)	0.0154 (6)	0.0210 (6)	−0.0019 (5)	0.0015 (5)	0.0003 (5)
C23	0.0251 (7)	0.0179 (7)	0.0236 (7)	−0.0022 (5)	0.0037 (5)	0.0032 (5)
C8	0.0248 (7)	0.0253 (7)	0.0338 (8)	0.0065 (6)	−0.0008 (6)	0.0030 (6)
C22	0.0336 (8)	0.0199 (7)	0.0286 (7)	−0.0052 (6)	0.0033 (6)	−0.0057 (6)
C17	0.0253 (6)	0.0176 (6)	0.0168 (6)	0.0032 (5)	0.0003 (5)	0.0014 (5)
C13	0.0336 (8)	0.0194 (7)	0.0328 (8)	0.0022 (6)	0.0040 (6)	0.0046 (6)
C9	0.0305 (8)	0.0345 (9)	0.0298 (7)	0.0155 (7)	0.0038 (6)	0.0080 (7)
C5	0.0196 (6)	0.0255 (7)	0.0275 (7)	−0.0007 (6)	−0.0011 (5)	−0.0086 (6)
C18	0.0205 (6)	0.0256 (7)	0.0186 (6)	0.0047 (6)	0.0058 (5)	0.0033 (5)
C11	0.0265 (7)	0.0315 (8)	0.0267 (7)	0.0098 (6)	−0.0050 (5)	−0.0075 (6)
C24	0.0216 (7)	0.0204 (7)	0.0349 (8)	−0.0012 (6)	−0.0027 (5)	0.0016 (6)
C10	0.0349 (8)	0.0378 (9)	0.0238 (7)	0.0176 (7)	−0.0036 (6)	−0.0032 (7)
C12	0.0319 (8)	0.0247 (7)	0.0339 (8)	0.0047 (6)	0.0043 (6)	−0.0062 (7)
C7	0.0230 (7)	0.0253 (7)	0.0248 (7)	0.0042 (6)	−0.0016 (5)	−0.0021 (6)

### Geometric parameters (Å, °)

**Table:**

O1—C2	1.4243 (17)	C23—H23C	0.98
O1—C5	1.4441 (17)	C8—C7	1.388 (2)
O20—C15	1.3638 (15)	C8—C9	1.391 (2)
O20—H20	0.90 (2)	C8—H8	0.95
N3—C13	1.4667 (19)	C22—H22A	0.98
N3—C2	1.4756 (18)	C22—H22B	0.98
N3—C4	1.4805 (18)	C22—H22C	0.98
C16—C17	1.3970 (18)	C17—C18	1.393 (2)
C16—C15	1.4151 (18)	C17—H17	0.95
C16—C21	1.5327 (18)	C13—H13A	0.98
C14—C19	1.3911 (18)	C13—H13B	0.98
C14—C15	1.4064 (18)	C13—H13C	0.98
C14—C2	1.5081 (19)	C9—C10	1.387 (3)
C19—C18	1.385 (2)	C9—H9	0.95
C19—H19	0.95	C5—H5	1
C2—H2	1	C18—H18	0.95
C6—C11	1.396 (2)	C11—C10	1.389 (2)
C6—C7	1.397 (2)	C11—H11	0.95
C6—C5	1.515 (2)	C24—H24A	0.98
C4—C12	1.516 (2)	C24—H24B	0.98
C4—C5	1.548 (2)	C24—H24C	0.98
C4—H4	1	C10—H10	0.95
C21—C22	1.5338 (19)	C12—H12A	0.98
C21—C24	1.540 (2)	C12—H12B	0.98
C21—C23	1.5433 (18)	C12—H12C	0.98
C23—H23A	0.98	C7—H7	0.95
C23—H23B	0.98		
			
C2—O1—C5	108.87 (11)	C21—C22—H22B	109.5
C15—O20—H20	106.2 (13)	H22A—C22—H22B	109.5
C13—N3—C2	111.67 (11)	C21—C22—H22C	109.5
C13—N3—C4	113.71 (12)	H22A—C22—H22C	109.5
C2—N3—C4	102.96 (11)	H22B—C22—H22C	109.5
C17—C16—C15	116.81 (12)	C18—C17—C16	122.63 (13)
C17—C16—C21	122.37 (12)	C18—C17—H17	118.7
C15—C16—C21	120.75 (11)	C16—C17—H17	118.7
C19—C14—C15	119.83 (12)	N3—C13—H13A	109.5
C19—C14—C2	118.96 (12)	N3—C13—H13B	109.5
C15—C14—C2	121.05 (11)	H13A—C13—H13B	109.5
C18—C19—C14	120.22 (13)	N3—C13—H13C	109.5
C18—C19—H19	119.9	H13A—C13—H13C	109.5
C14—C19—H19	119.9	H13B—C13—H13C	109.5
O1—C2—N3	103.56 (10)	C10—C9—C8	119.59 (15)
O1—C2—C14	109.16 (12)	C10—C9—H9	120.2
N3—C2—C14	114.06 (11)	C8—C9—H9	120.2
O1—C2—H2	110	O1—C5—C6	108.80 (12)
N3—C2—H2	110	O1—C5—C4	104.91 (11)
C14—C2—H2	110	C6—C5—C4	117.42 (11)
C11—C6—C7	119.09 (14)	O1—C5—H5	108.5
C11—C6—C5	119.00 (14)	C6—C5—H5	108.5
C7—C6—C5	121.90 (13)	C4—C5—H5	108.5
O20—C15—C14	120.21 (12)	C19—C18—C17	119.46 (12)
O20—C15—C16	118.81 (12)	C19—C18—H18	120.3
C14—C15—C16	120.97 (11)	C17—C18—H18	120.3
N3—C4—C12	112.88 (12)	C10—C11—C6	120.52 (15)
N3—C4—C5	101.85 (11)	C10—C11—H11	119.7
C12—C4—C5	116.12 (13)	C6—C11—H11	119.7
N3—C4—H4	108.5	C21—C24—H24A	109.5
C12—C4—H4	108.5	C21—C24—H24B	109.5
C5—C4—H4	108.5	H24A—C24—H24B	109.5
C16—C21—C22	112.05 (11)	C21—C24—H24C	109.5
C16—C21—C24	111.12 (11)	H24A—C24—H24C	109.5
C22—C21—C24	107.05 (12)	H24B—C24—H24C	109.5
C16—C21—C23	108.84 (11)	C9—C10—C11	120.18 (15)
C22—C21—C23	107.38 (12)	C9—C10—H10	119.9
C24—C21—C23	110.34 (11)	C11—C10—H10	119.9
C21—C23—H23A	109.5	C4—C12—H12A	109.5
C21—C23—H23B	109.5	C4—C12—H12B	109.5
H23A—C23—H23B	109.5	H12A—C12—H12B	109.5
C21—C23—H23C	109.5	C4—C12—H12C	109.5
H23A—C23—H23C	109.5	H12A—C12—H12C	109.5
H23B—C23—H23C	109.5	H12B—C12—H12C	109.5
C7—C8—C9	120.51 (16)	C8—C7—C6	120.11 (14)
C7—C8—H8	119.7	C8—C7—H7	119.9
C9—C8—H8	119.7	C6—C7—H7	119.9
C21—C22—H22A	109.5		
			
C15—C14—C19—C18	−0.3 (2)	C17—C16—C21—C24	−121.31 (14)
C2—C14—C19—C18	175.24 (12)	C15—C16—C21—C24	61.85 (16)
C5—O1—C2—N3	−29.88 (13)	C17—C16—C21—C23	116.99 (14)
C5—O1—C2—C14	−151.74 (11)	C15—C16—C21—C23	−59.85 (16)
C13—N3—C2—O1	164.76 (12)	C15—C16—C17—C18	1.39 (19)
C4—N3—C2—O1	42.40 (13)	C21—C16—C17—C18	−175.57 (12)
C13—N3—C2—C14	−76.72 (15)	C7—C8—C9—C10	0.9 (2)
C4—N3—C2—C14	160.91 (11)	C2—O1—C5—C6	132.44 (12)
C19—C14—C2—O1	−95.64 (14)	C2—O1—C5—C4	6.00 (14)
C15—C14—C2—O1	79.84 (15)	C11—C6—C5—O1	142.27 (13)
C19—C14—C2—N3	149.09 (13)	C7—C6—C5—O1	−36.26 (17)
C15—C14—C2—N3	−35.44 (17)	C11—C6—C5—C4	−98.86 (15)
C19—C14—C15—O20	−178.14 (12)	C7—C6—C5—C4	82.61 (17)
C2—C14—C15—O20	6.42 (19)	N3—C4—C5—O1	19.92 (13)
C19—C14—C15—C16	2.55 (19)	C12—C4—C5—O1	142.96 (13)
C2—C14—C15—C16	−172.88 (13)	N3—C4—C5—C6	−101.00 (13)
C17—C16—C15—O20	177.65 (12)	C12—C4—C5—C6	22.04 (18)
C21—C16—C15—O20	−5.34 (18)	C14—C19—C18—C17	−1.3 (2)
C17—C16—C15—C14	−3.03 (18)	C16—C17—C18—C19	0.8 (2)
C21—C16—C15—C14	173.98 (12)	C7—C6—C11—C10	0.3 (2)
C13—N3—C4—C12	76.22 (16)	C5—C6—C11—C10	−178.31 (13)
C2—N3—C4—C12	−162.80 (12)	C8—C9—C10—C11	−0.8 (2)
C13—N3—C4—C5	−158.56 (12)	C6—C11—C10—C9	0.2 (2)
C2—N3—C4—C5	−37.58 (13)	C9—C8—C7—C6	−0.4 (2)
C17—C16—C21—C22	−1.61 (18)	C11—C6—C7—C8	−0.2 (2)
C15—C16—C21—C22	−178.45 (12)	C5—C6—C7—C8	178.33 (13)

### Hydrogen-bond geometry (Å, °)

**Table:**

*D*—H···*A*	*D*—H	H···*A*	*D*···*A*	*D*—H···*A*
O20—H20···N3	0.90 (2)	1.79 (2)	2.6244 (16)	154.4 (19)
